# Validation of a novel 3D-printed anthropomorphic pediatric abdomen phantom using photon-counting CT

**DOI:** 10.1038/s41598-025-32391-2

**Published:** 2025-12-12

**Authors:** Thilo Schikorra, Matthias S. May, Thomas G. Flohr, Thomas Allmendinger, Ferdinand Knieling, Ido Bitan, Reut Reina, Michael Uder, Markus Kopp

**Affiliations:** 1 Department of Radiology, University Hospital Erlangen, Friedrich-Alexander-Universität Erlangen-Nürnberg (FAU), 91054 Erlangen, Germany; 2 Siemens Healthineers GmbH, 91301 Forchheim, Germany; 3 Department of Paediatrics and Adolescent Medicine, Friedrich-Alexander-Universität Erlangen-Nürnberg (FAU), 91054 Erlangen, Germany; 4 Stratasys Ltd., 7612401 Rehovot, Israel

**Keywords:** 3D-printed phantom, Anthropomorphic phantom, Photon-counting CT, Pediatric imaging, CT dose optimization, Image quality assessment, Medical imaging, Paediatrics, Biological physics, Paediatric research, Preclinical research

## Abstract

This study compares objective and subjective image quality (IQ) between a pediatric patient’s CT scan and a subsequently 3D-printed anthropomorphic abdomen CT phantom on a photon-counting CT (PCCT). We used a dedicated 3D-printed CT phantom based on DICOM data obtained from a PCCT scan of a 5-year-old boy (70 kV, 458 mAs). We performed two scans on the phantom (1: 70 kV, 461 mAs and 2: 90 kV, 148 mAs), reconstructing them with three kernels. We measured the objective IQ based on mean CT values and image noise within eight target structures. Three radiology experts assessed the subjective IQ on a 5-point Likert scale. The CT values of organs and vessels in phantom scan 1 (Br40) were equivalent to those in the patient (*p* = 0.019). The phantom demonstrated higher mean CT values for fat and lower CT values for bone. The image noise was equivalent between both scans (*p* = 0.027). The phantom showed high subjective IQ but a significantly lower rating than the patient (median 4 vs. 5, *p* < 0.001). In conclusion, this study demonstrates comparable IQ between the phantom and the patient scan at comparable dose parameters. In the future, it may be possible to anticipate radiation exposure in dose reduction studies.

## Introduction

Computed tomography (CT) is essential for diagnostic workup and therapy planning in a variety of diseases. However, CT scans are still a relevant source of radiation, particularly for children, who generally show a high vulnerability to radiation dose and, additionally, have a longer life expectancy, meaning a higher probability of developing cancerous lesions caused by DNA damage ^1–3^. Therefore, the radiation dose must be kept as low as reasonably achievable (ALARA principle) with respect to the linear non-threshold thesis regarding stochastic radiation risk. It remains an important task to reduce the radiation dose further while maintaining sufficient image quality ^4^.

Because of their diagnostic value, speed, non-invasiveness, and high availability, the frequency of CT scans has increased worldwide, from 34 scans per 1,000 people per year in 2006 to 55 in 2018 ^5^. The development of low-dose protocols and new scan technologies in CT and other radiation-emitting diagnostic and therapeutic procedures ^6,7^ could reduce the annual average effective dose per person worldwide from 0.65 to 0.56 mSv ^8^. Despite the higher vulnerability of pediatric patients to low-dose radiation exposure, many pediatric diseases require three-dimensional CT imaging for accurate diagnosis ^9–13^.

To decrease radiation exposure, continuously improved scanner technology is essential. Novel photon-counting CT (PCCT) technology is the most recent approach, which is highly dose-effective due to its direct conversion of X-rays into an electrical signal^14–16^. However, further dose reduction studies are required to determine the lowest clinically sufficient radiation dose for specific examinations. Currently, such studies are limited or even impossible because recurrent CT scans of patients at different dose levels cannot be approved due to ethical concerns.

An anthropomorphic 3D phantom potentially enables a more realistic and repeatable analysis of image quality as a function of radiation exposure. Corresponding phantoms have undergone continuous improvement, from simple oval-shaped models made from a single tissue-equivalent material in 1986^17^, to 3D-printed phantoms with varied shapes made from multiple uniform materials in 2005^18^, to even more realistic phantoms created using consecutive layers of paper-printed with radiopaque ink in 2016^19^. However, even the latest available phantoms have some limitations. They are not entirely anatomically comparable to patients’ CT scans; they misrepresent CT values, and most of them have a limited resolution, mechanical instability, and low reproducibility due to their manufacturing procedure. Recently, however, these challenges have been addressed with the presentation of a realistic 3D-printed cervical vertebrae phantom, a knee phantom^20^, and a 3D-printed slice of a head^21^.

Building on these developments, we created a dedicated 3D-printed anthropomorphic pediatric abdomen phantom using the data from a pediatric CT scan (StrataConv, Siemens Healthineers, Forchheim, Germany; Voxel Print, Stratasys Ltd., Rehovot, Israel). This study aims to test the null hypothesis that the image quality of the 3D abdomen CT phantom and patient scan is not comparable in terms of objective and subjective image quality.

## Materials and methods

### Patients

We used anonymized CT raw data from a portal venous abdominal CT scan of a 5-year-old boy, who was scanned as part of routine clinical practice with a dual-source photon-counting CT (Naeotom Alpha, Siemens Healthineers, Forchheim, Germany). The scan parameters were as follows: 70 kV tube voltage, IQ-level 72 (a company-specific metric that adjusts the radiation dose based on the patient size derived from the topogram and scanner-specific characteristics adapted to the clinical scan to ensure consistent imaging performance), with automatic exposure control (CAREDose4D, Siemens Healthineers), 458 quality reference mAs (qRef mAs), detector collimation 144 × 0.4 mm, gantry rotation time 0.25 s, and pitch 2.4 Dual Source scan. The volume computed tomography dose index (CTDI_vol_), as indicated by the automatic dose reporting system of the CT scanner, was 1.14 mGy. We reconstructed polychromatic T3D images at a slice width of 3 mm, with an increment of 3 mm, employing kernels Br40, Br60, and Quantum Iterative Reconstruction (QIR) level 3. Subsequently, the data underwent anonymization. (Table 1)

The study complies with the Declaration of Helsinki. The patient scan was acquired in a routine clinical setting following a justifying medical indication. The scan protocol followed institutional standard operational procedures. No additional radiation exposure or modification for study purposes were necessary. The radiation dose level of 1.14 mGy CTDI_vol_ in the patient scan is below the limits of the German Federal Office for Radiation Protection of 4.0 mGy^22^. The image data does not include any identifying patient or imaging information.

**Table 1:** Scan parameters and dose levels for the patient and phantom scans and corresponding reconstruction parameters.

Scans	Tube voltage [kV]	qRef mAs [mAs]	CTDI_vol_ [mGy]	Spiral pitch factor	Reconstruction kernel	Slice width [mm]
Patient	70	458	1.14	2.4	Br40 Br60	0.6 & 3.0
Phantom	70	461	0.97	2.4	Br40 Br44 Br60	0.6 & 3.0
Phantom	90	148	1.07	2.4	Br40 Br60	0.6 & 3.0

### 3D-printed abdominal CT phantom

**Fig. 1:**
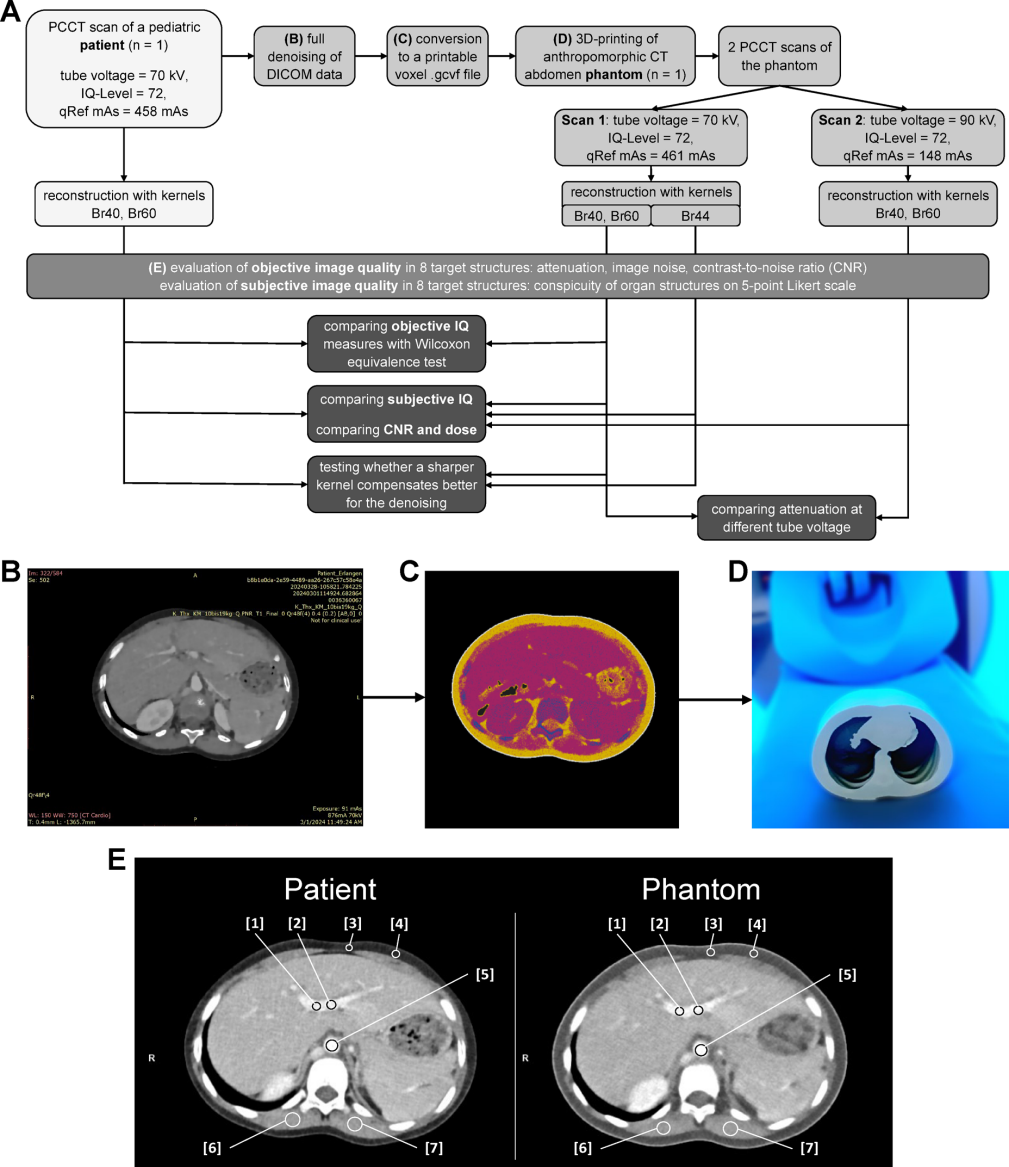
Overview of the methods of the presented study. (**A**) Flow chart: We obtained image data from one photon-counting CT (PCCT) scan of a pediatric patient. After reconstructing, fully denoising, and converting the DICOM data to a printable volumetric pixel (voxel) file, the CT abdomen phantom was 3D-printed (B-D). We performed two PCCT scans on the phantom. Scan 1 has scan parameters comparable to the patient scan: tube voltage = 70 kV, Image Quality Level = 72, and quality Reference mAs (qRef mAs) = 461 mAs. Scan 2 has the same parameters but tube voltage = 90 kV and qRef mAs = 148 mAs. We reconstructed patient and phantom scans with Br40 and Br60. When designing the study, we planned to reconstruct scan 1 of the phantom additionally with Br44. For all reconstructions, we evaluated the objective image quality (IQ). We compared the objective and subjective IQ of the Br40 reconstructions of phantom scan 1 and the patient scan. We expected slightly blurrier images due to the extensive denoising in the production process of the phantom. Consequently, we tested whether the sharper Br44 reconstruction of the phantom compensates more effectively for denoising. We also compared the attenuation of different tissues between scan 1 and scan 2 to evaluate the spectral properties of the phantom. (**B**) Fully denoised image of the patient scan: A prototype reconstruction algorithm provided maximum image denoising to reduce the negative influence of noise on the phantom quality. (**C**) Converted three-color image of the patient scan: Each color represents the CT value of one of the photopolymer printing materials used. (**D**) Macroscopic morphology of the 3D-printed abdomen CT phantom in the PCCT (**E**) Exemplary positioning of seven circular regions of interest (ROIs) in an axial image slice of the patient scan (70 kV, Br40) and in similar positions in the phantom scan (70 kV, Br40): To evaluate objective image quality, we placed ROIs in different tissues. The images show ROIs in the portal vein [1, 2], abdominal fat [3, 4], aorta [5], and autochthonous muscle [6, 7] as representative examples.

The present study utilizes a 3D printing solution based on PolyJet™ multi-material photopolymer 3D printing technology (J850™ Digital Anatomy™, Stratasys Ltd., Rehovot, Israel) from a single vendor (Stratasys Ltd.). It is based on a pixel-to-pixel mapping process between clinical CT images and three polymer printer materials that exhibit different radiopacities (StrataConv, Siemens Healthineers, Forchheim, Germany) as well as software that creates the printable voxel file (Voxel Print, Stratasys Ltd.). A flow chart of the study is provided in Fig. 1A.

As the initial step, the anonymized raw data from the scan was transferred to an offline workstation for a dedicated image reconstruction. Derived from the PCCT product iterative reconstruction (Quantum Iterative Reconstruction, Siemens Healthineers, Germany), an innovative prototype version was applied, which maximizes the image denoising process. Image reconstruction parameters were Qr48, 0.4 mm slice width, 0.2 mm increment, field of view 220 mm, matrix 512 × 512. The primary aim of this process is to create completely noise-free images, accepting a certain loss of object sharpness or small detail. To our knowledge, this is the first published approach to apply this extensive denoising to the CT data prior to printing. The method is based on the idea that a substantial noise texture contribution, if printed into the phantom, would systematically influence the realized noise power spectrum of the scanned phantom and, therefore, the image impression in terms of noise and texture. Only phantoms with a correct noise power spectrum exhibit a correct ratio between dose and image noise, which is mandatory for dose reduction studies. Figure 1B provides an example of these fully denoised images.

In a second step, these images were transformed by dedicated internal prototype software (StrataConv, Siemens Healthineers) for a pixel-to-pixel mapping process between the CT images and three photopolymer printer materials, which exhibit different radiopacities. These three materials realize support points with a fixed Hounsfield value for a given x-ray spectrum in the resulting phantom-derived images with calibrated values of -30 HU (TissueMatrix^®^), 100 HU (Vero^®^ClearB), and 1400 HU (RadioMatrix™) at 70 keV. These HU values are derived from internal calibrations performed by Siemens using cylinders composed of 100% target material. All intermediate Hounsfield values were generated by selecting these materials in relative fractions based on material pairs along the full dynamic CT scale in a dithering approach. The software subsequently utilizes the high in-plane resolution of the 3D printer of 600 × 300 dpi, in combination with a slice thickness of 27 μm (J850™ Digital Anatomy™, Stratasys Ltd.), which exceeds the achievable resolution of the CT system in pediatric abdominal imaging.

As a result of the steps described above, a stack of 4,398 bitmap three-color images representing the individual materials by color was created in a resolution of 5905 × 2952 (see Fig. 1C). With dedicated software (Voxel Print, Stratasys Ltd.), the PNG images were converted to a printable voxel (volumetric pixel) .gcvf file. For printing the model, the J850™ Digital Anatomy™ printer (Stratasys Ltd., Rehovot, Israel) used the above-mentioned materials TissueMatrix^®^, Vero^®^ClearB, and RadioMatrix™ as well as a support material (GelMatrix^®^) for small internal cavities, which can be easily removed with a water jet. The process resulted in the final 3D-printed anthropomorphic pediatric abdomen CT phantom shown in Fig. 1D.

### Phantom evaluation

For the PCCT scans of the anthropomorphic pediatric abdomen CT phantom, we used the same scan parameters as for the patient scan and selected the same IQ level of 72, corresponding to 461 qRef mAs. Additionally, we performed a CT scan at a different tube voltage (90 kV) but with the same IQ level (72), corresponding to 148 qRef mAs. The CTDI_vol_ was assessed using the scanner’s automated dose reporting system. (see Table 1)

We reconstructed polychromatic T3D images of each scan with a Quantum Iterative Reconstruction (QIR) of 3, a slice thickness of 3 mm, an increment of 1 mm, and kernels Br40 and Br60. For the 70 kV scan, we performed an additional reconstruction with a sharper Br44 kernel to compensate for a potential blurring of structures in the phantom printing process. In addition to the slice width of 3 mm, we reconstructed images with a slice width of 0.6 mm for coronal reformations of the phantom scans (see Table 1).

We compared the CT scans of the phantom reconstructed with Br40 and Br44 to the patient scan regarding several image quality metrics. We analyzed the following objective parameters: (1) attenuation in regions of interest (ROI) in different tissues, (2) image noise in ROIs in different tissues, and (3) contrast-to-noise ratio (CNR). Additionally, we evaluated the subjective image quality of different anatomical structures. Furthermore, the phantom scan at 70 kV was compared to the 90 kV scan to evaluate spectral properties at different tube voltages.

#### Objective image quality evaluation

Using a dedicated 3D-imaging software (Syngo.Via VB80E, Siemens Healthineers, Forchheim, Germany) we measured attenuation, image noise and CNR in circular ROIs within the following target structures: (1) liver-parenchyma, (2) portal vein, (3) abdominal aorta, (4) abdominal fat, (5) autochthonous dorsal muscles, (6) spleen, (7) pancreas, and (8) ribs. We placed ROIs in different locations of each of the target structures (*n* = 8) of the patient CT scan (458 qRef mAs, Br40), including only homogenous tissue without vessels or lesions. We selected the number of ROIs and size in cm² depending on the target’s organ size and shape:

- (1) liver-parenchyma: 4 ROIs (1.50 cm²).
- (2) V. portae: 2 ROIs (0.25 cm²).
- (3) abdominal aorta: 2 ROIs (1 × 0.50 cm² & 1 × 0.20 cm²).
- (4) dorsal & ventral abdominal fat: 4 ROIs (2 × 2.00 cm² & 2 × 0.20 cm²).
- (5) autochthonous dorsal muscles: 4 ROIs (0.60 to 0.80 cm²).
- (6) spleen: 3 ROIs (1.00 cm²).
- (7) pancreas: 3 ROIs (1.10 cm²).
- (8) ribs: 4 ROIs (0.01 cm²).

We repeated this procedure for each of the performed CT scans and reconstructions of the phantom (70 kV and 90 kV, for reconstruction kernels Br40, Br44), taking care to place ROIs of the same size at the same location, orientated at anatomical landmarks (see Fig. 1E). We evaluated 26 ROIs per reconstruction, in total 104 ROIs.

We defined attenuation as the mean CT values (in Hounsfield Units HU) of the target ROI and image noise as the standard deviation (SD) of the CT values. We did not calculate the image noise of the ribs as the measuring of image noise in solid bone is inconclusive due to the small ROI size. The contrast-to-noise ratio (CNR) was calculated as the difference in CT values (CT) between portal vein and liver, or between aorta and muscle, divided by the background image noise (SD) in liver parenchyma or muscle:

$$\:\text{C}\text{N}\text{R}=\frac{{\text{C}\text{T}}_{\text{t}\text{i}\text{s}\text{s}\text{u}\text{e}}\:-\:{\text{C}\text{T}}_{\text{b}\text{a}\text{c}\text{k}\text{g}\text{r}\text{o}\text{u}\text{n}\text{d}}}{{\text{S}\text{D}}_{\text{b}\text{a}\text{c}\text{k}\text{g}\text{r}\text{o}\text{u}\text{n}\text{d}}}$$

### Subjective image quality evaluation

We performed a subjective image quality evaluation for the following eight target regions:

- (1) delineation between the renal cortex and medulla.
- (2) delineation between the gallbladder and abdominal fat tissue.
- (3) delineation between the pancreas and abdominal fat tissue.
- (4) delineation between an arterial liver hyperenhancement in segments II/III and liver parenchyma.
- (5) sharpness of the posterior right portal venous branch between segments VI and VII against liver parenchyma.
- (6) sharpness of the splenic vein against abdominal fat tissue.
- (7) sharpness of the cortex of the vertebral body L1.
- (8) sharpness of bone matrix structures in the vertebral body L1.

For reasons of consistency, we specifically selected the posterior right portal venous branches between liver segments VI and VII (criterion 5) and the vertebral body of L1 (criteria 7 and 8). The adrenal glands were not selected as target structures, as they are not sufficiently definable in the patient scan. The criteria were rated on a 5-point Likert scale:

- 1: no delineation/borderline visible.
- 2: very blurry delineation/structure.
- 3: slightly blurry delineation/structure.
- 4: good delineation/sharpness.
- 5: perfect delineation/sharpness.

Three trained radiology experts with 30, 11, and 1 year of experience evaluated the subjective overall image quality of each of the target structures in the axial reconstructions at a fixed window setting (W:470 C:40). The bone structures were rated in the Br60 reconstruction following clinical practice, using a typical bone window (W:3000, C:1000). We held a preparatory session, in which we showed exemplary images for each Likert scale rating to ensure consistent ratings.

### Statistical tests

We paired the CT values of the ROIs in the patient scan with the corresponding CT values of the Br40 phantom scan reconstruction and statistically analyzed them. Given the sample size of *n* = 26 CT values per scan and the requirement of testing paired data for equivalence, we selected the non-parametric Wilcoxon signed-rank test for equivalence with α = 0.05 as a non-parametric alternative to the TOST (two one-sided t-tests)^23^. For the test, it is necessary to select upper and lower boundaries as clinically acceptable values for equivalence. A review of literature failed to yield any studies that define such boundaries for CT values of phantoms. Consequently, we used studies on diagnostic HU thresholds and inter-scanner variability to estimate a limit for our study. In clinical practice, the use of differences in absolute CT values is a widely accepted approach for distinguishing specific lesions. A threshold between 10 and 16 HU is generally accepted for detecting adrenal adenomas with high sensitivity^24,25^. An enhancement of 10 HU has been identified as a threshold value for detecting pseudoenhancing renal cysts^26^. The variability between CT scanners of different vendors ranged from 7 to 56 HU for different tested materials in one study^27^ and from 9 to 10 HU for a 0 HU material in another study^28^. Consequently, we defined a limit of ± 6.0 HU as the boundary for the equivalence test, which is more than 10% lower than the lowest reported inter-scanner variability and within the thresholds used in clinical practice. To analyze differences between different types of structures in the phantom, we performed two tests: (1) comparing organs, muscle, and vessels but excluding ribs and fat to evaluate the HU-realism of the phantom in tissues with average CT values of 50 to 300 HU, (2) comparing only fat and rib bone to evaluate very low and high CT values.

We calculated the average and SD of the image noise for each tissue in the scans and reconstructions. We paired the values of the image noise in the patient scan with the corresponding values of the Br40 reconstruction of the phantom scan and statistically tested for equivalence. For the same reasons as listed above (*n* = 26 SDs per scan, paired data), we selected the equivalence test based on Wilcoxon, with α = 0.05 and clinically acceptable boundaries. As we have not found studies defining such an acceptable level of deviation in image noise, we estimated a limit based on inter-scanner variability studies. Montero et al. reported a coefficient of variation of 10.9% for image noise across different CT systems^29^. Assuming an average noise level of 15 to 25 HU in current low-dose CT^30,31^, this corresponds to a variability of 1.6 to 2.7 HU. Given these findings, we defined acceptable image noise boundaries of ± 1.4 HU, which is more than 10% lower than the observed inter-scanner variability.

Additionally, we compared the differences in the image noise between the patient and phantom scans (Br40, Br44) using a paired Wilcoxon signed-rank test. This analysis aimed to evaluate whether a sharper reconstruction kernel provides a closer approximation to the patient scan in terms of image noise.

In this study, a p-value < 0.05 was considered an acceptable level of statistical significance. The Wilcoxon signed-rank equivalence test includes two tests for the stated lower and upper boundary. Since both tests need to be significant for the two compared data sets to be considered statistically equivalent within the boundaries, the maximum p-value determines the result. We performed the Wilcoxon signed-rank test for equivalence using the wilcox_TOST function of R Statistical Software (v4.4.2; R Core Team 2024, Vienna, Austria) and the TOSTER R package (v0.8.3)^32,33^. The Wilcoxon test was performed using IBM SPSS Statistics for Windows (version 29.0.1.0, IBM Corp., 2023, Armonk, New York).

For the subjective rating, the inter-rater reliability (IRR) among the three raters was assessed using the two-way mixed, absolute, average-measures intraclass correlation coefficient (ICC)^34^. Following commonly cited cutoffs^35^, ICC values above 0.75 indicate excellent agreement. We calculated the median, minimal, and maximal Likert scores for each organ and scan. As the ratings on the 5-point Likert scale are ordinal and the sample size is small (*n* = 24 ratings per scan), we chose the Wilcoxon signed-rank test for paired comparisons of the different scan reconstructions. We conducted three tests: (1) patient vs. phantom (70 kV, Br40) to evaluate whether the phantom scan was rated different than the patient scan, (2) phantom (70 kV, Br40) vs. phantom (70 kV, Br44) to compare two reconstruction kernels, and (3) phantom (70 kV, Br40) vs. phantom (90 kV) to compare two tube voltages. We performed all tests and calculations for the subjective rating in the software IBM SPSS Statistics for Windows.

## Results

### Evaluation of CT scans

#### Objective image quality evaluation

**Fig. 2:**
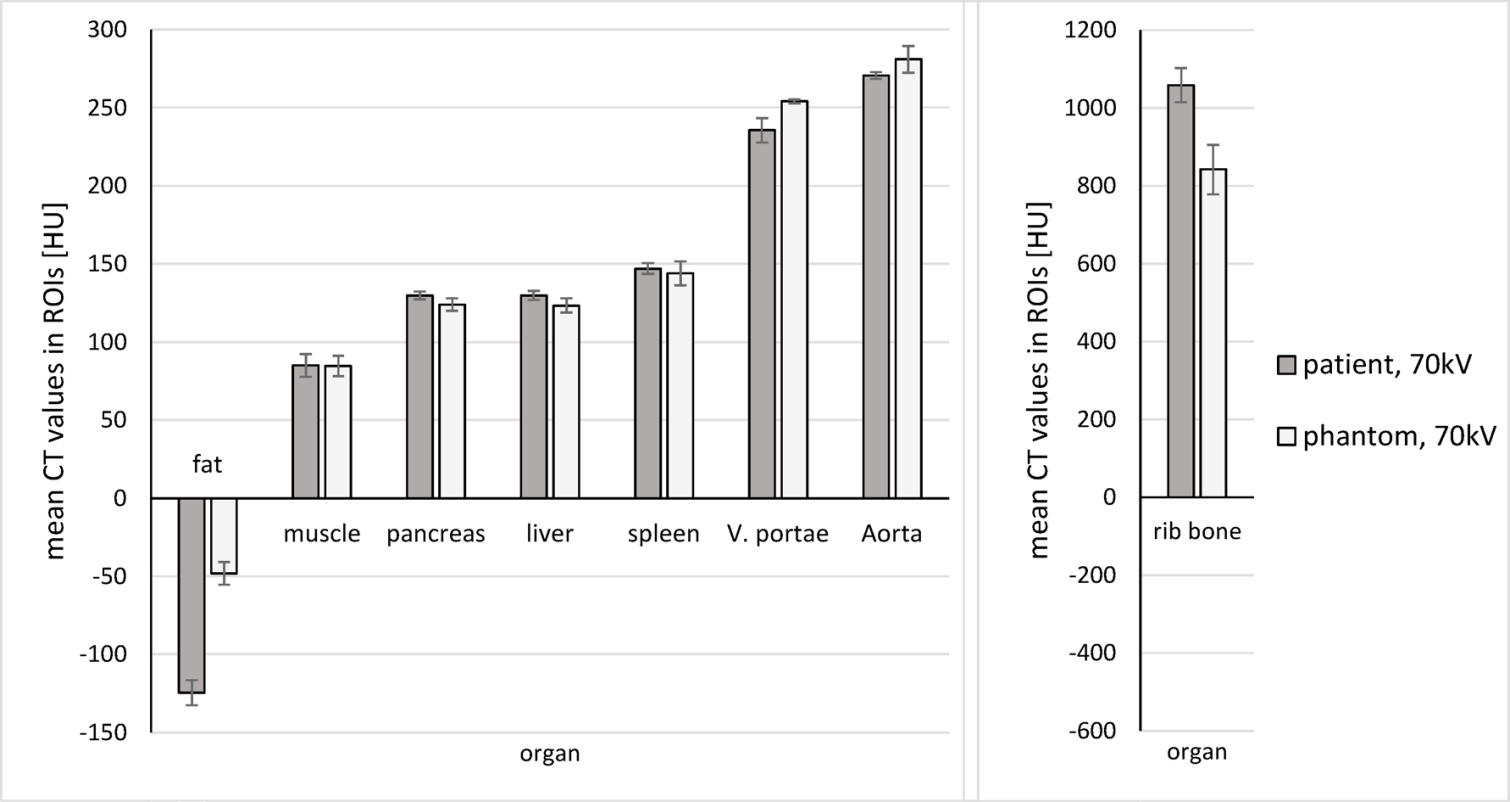
CT values of eight target structures in the patient and phantom scans: We calculated the mean CT values (in HU) and standard deviations from regions of interest placed in the patient scan (70 kV, Br40) and the phantom scan (70 kV, Br40). The CT values are equivalent for organs, muscle tissue, and vessels (*p* = 0.019). We found no equivalence for fat and rib bone (*p* = 0.883).

CT values of the phantom scan at 70 kV (Br40) are equivalent for organs, muscle tissue, and vessels (*p* = 0.019). For fat and rib bone, we found no equivalence (*p* = 0.883). Fat shows higher mean CT values in the phantom than in the patient scan (-48.3 HU vs. -124.5 HU), while rib bone shows lower mean CT values (842.5 HU vs. 1059.5 HU). An illustration of mean CT values is presented in Fig. 2. The detailed results of the Wilcoxon-equivalence tests, testing for equivalence of CT values between the patient and the phantom scan (70 kV, Br40) with equivalence-boundary ± 6.0 HU are the following:

- CT values in organs, muscle, vessels: p_-6.0_ = 0.019, p_+ 6.0_ = 0.010 -> equivalent.
- CT values in fat and ribs p_-6.0_ = 0.147, p_+ 6.0_ = 0.883 -> not equivalent.

**Fig. 3:**
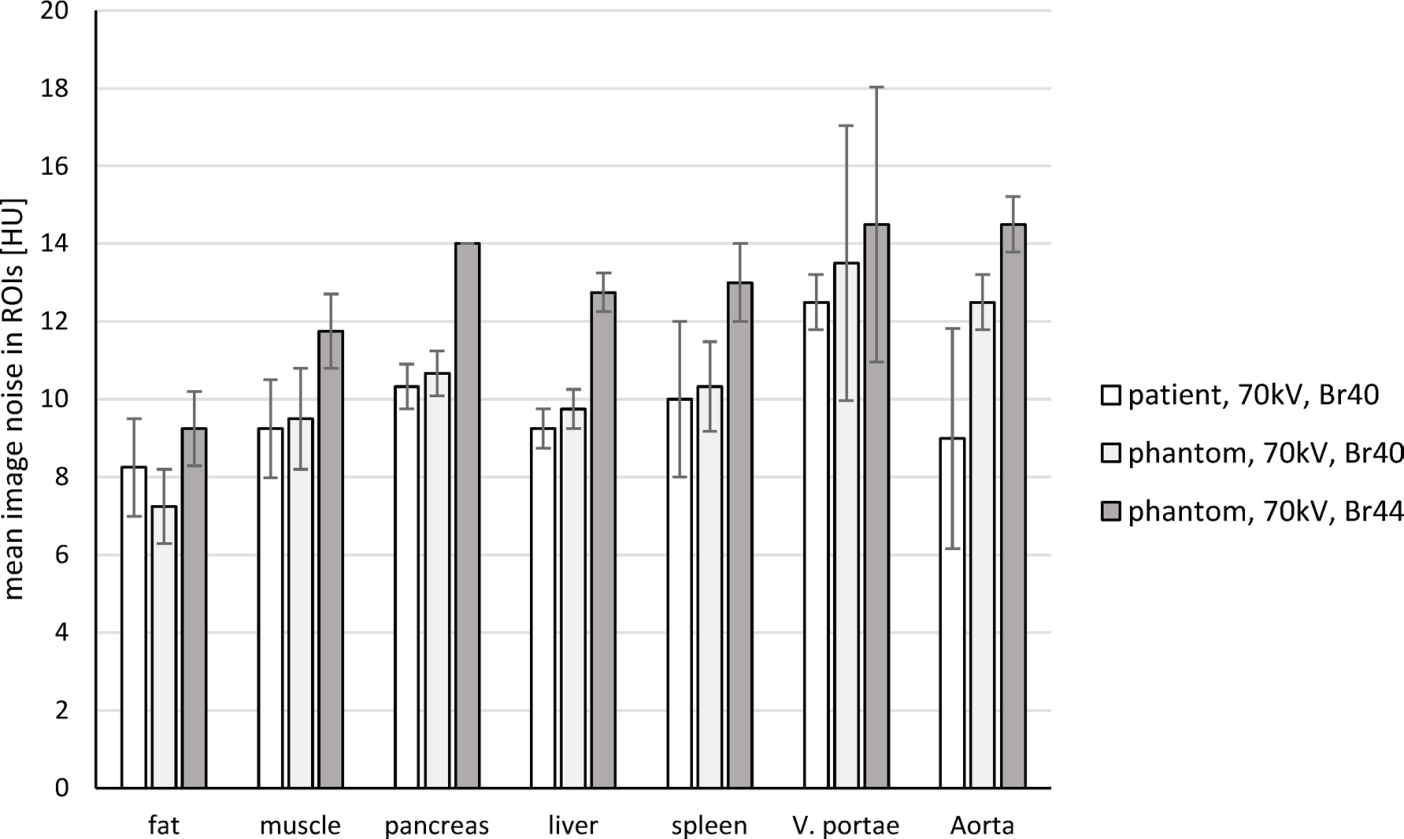
Image noise in various target structures in the patient and two phantom scan reconstructions: The graph shows the mean and standard deviation of the image noise in seven target structures in both the patient scan (70 kV, Br40) and the phantom scans (70 kV, Br40 & Br44). The image noise of the patient and phantom scan (Br40) is equivalent (*p* = 0.027). In the phantom scan reconstruction with Br44, the image noise increased (*p* < 0.001).

Image noise was found to be equivalent (*p* = 0.027) in all analyzed organs between the patient and phantom scans (70 kV, Br40). An illustration of the image noise in the target structures is shown in Fig. 3. In detail, the p-values of the Wilcoxon-equivalence tests testing for equivalence of image noise between all structures in the patient and the phantom scan (70 kV, Br40) with boundary ± 1.4 HU are: p_− 1.4_ = 0.027, p_+ 1.4_ = 0.001 -> equivalent.

The image noise in the Br44 reconstruction was higher than in the Br40 reconstruction in all evaluated organs. The paired Wilcoxon signed-rank test comparing absolute differences in image noise |Patient – Br40| to |Patient – Br44| showed significantly smaller deviations for Br40 (*p* < 0.001). This indicates that the Br40 reconstruction resembles the image noise of the patient scan better than the Br44 reconstruction. The image noise in target structures of the patient scan and the different phantom scan reconstructions is illustrated in Fig. 3.

**Fig. 4:**
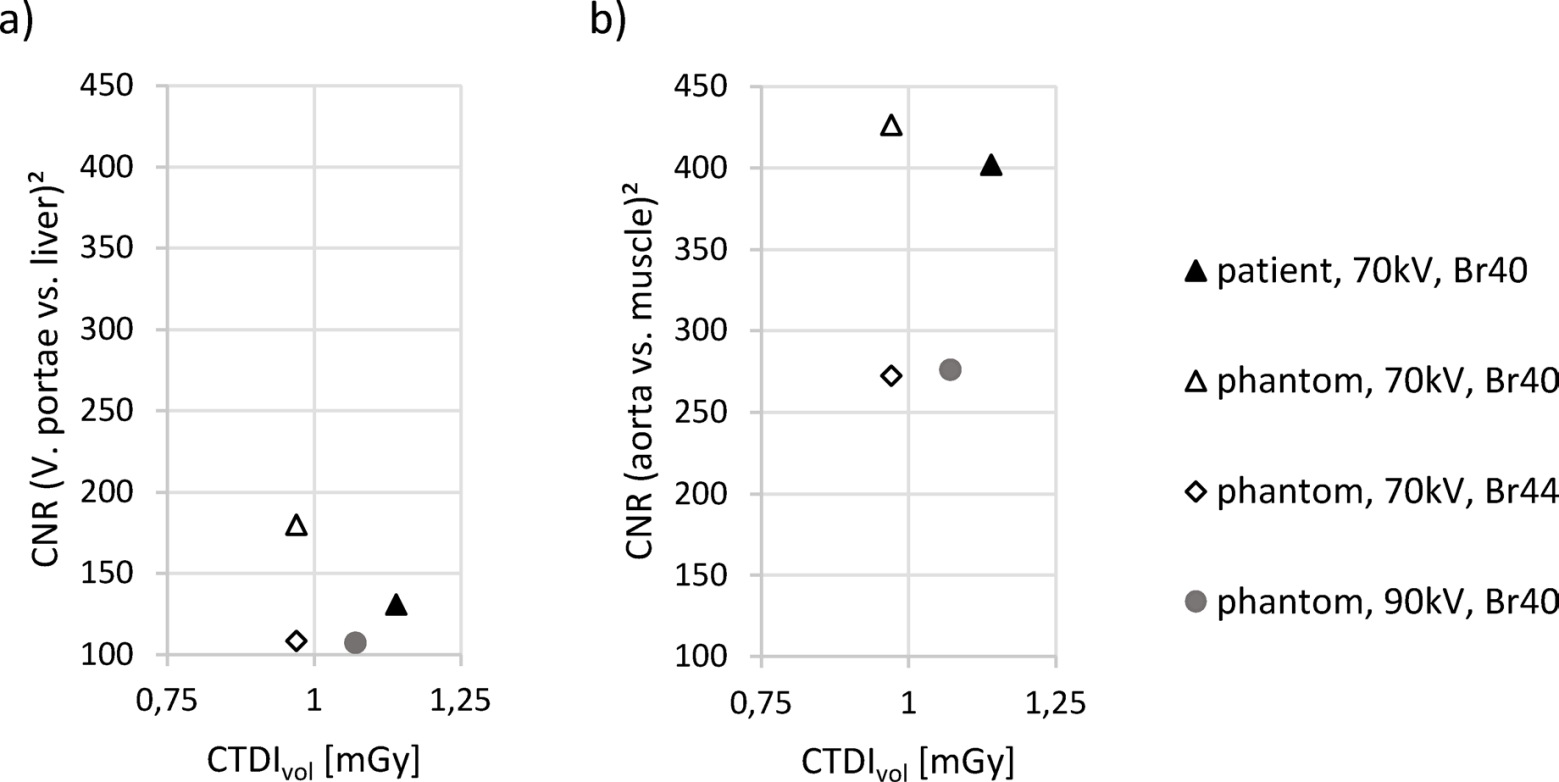
CNR^2^ for scans of the patient and phantom as a function of radiation dose measured as CTDI_vol_. (**a**) CNR of the V. portae with liver parenchyma as background-tissue. (**b**) CNR of the aorta with dorsal autochthonous muscle as background-tissue.

Evaluating the contrast, the CNR^2^ of the patient scan is lower than the CNR^2^ of the phantom scan (70 kV, Br40) at a similar radiation dose. CNR^2^ of the phantom scan at 90 kV is lower than at 70 kV. An illustration is provided in Fig. 4.

A comparison of the CT values of the phantom scans at 70 kV and 90 kV revealed that the average CT values of fat increased at higher tube voltage by 36.9% (-48.25 vs. -35.25 HU), while those of rib bone and the contrast-enhanced vessels portal vein and aorta decreased by 27.4%, 12.6%, and 11.7% (842.5 vs. 661, 254 vs. 225.5, 281 vs. 251.5 HU). The CT values of the parenchymatous organs muscle, pancreas, liver, and spleen increased in the 90 kV scan by 13.3%, 6.1%, 6.8%, and 2.7%.

### Subjective image quality evaluation

The patient scan was rated significantly better than the 70 kV Br40 phantom scan (median = 5 vs. 4; Z = -3.787; *p* < 0.001). The soft tissue structures in the phantom were consistently rated higher than the bone structures. Neither the sharper Br44 kernel (median = 4 vs. 4; Z = 0; *p* = 1.00) nor the 90 kV tube voltage (median = 4 vs. 4; Z = -1.633; *p* = 0.102) produced ratings that differed significantly from the 70 kV Br40 phantom scan.

The intra-class correlation of the subjective rating was in an excellent range (ICC = 0.84), indicating a high level of agreement between the three expert raters. For all structures in both the patient and the phantom scans, the median rating was at least 4 (clinically sufficient delineation and sharpness). The exceptions to this were the bone corticalis (criterion 6) and bone matrix structures (criterion 7), which were rated with a 5 (perfect sharpness) for the patient scan and a 3 (slightly blurry structure) for the phantom. In the phantom scan at 70 kV (Br40), the above-mentioned bone structures and the liver hyper-enhancement were the only items rated 3.

**Fig. 5:**
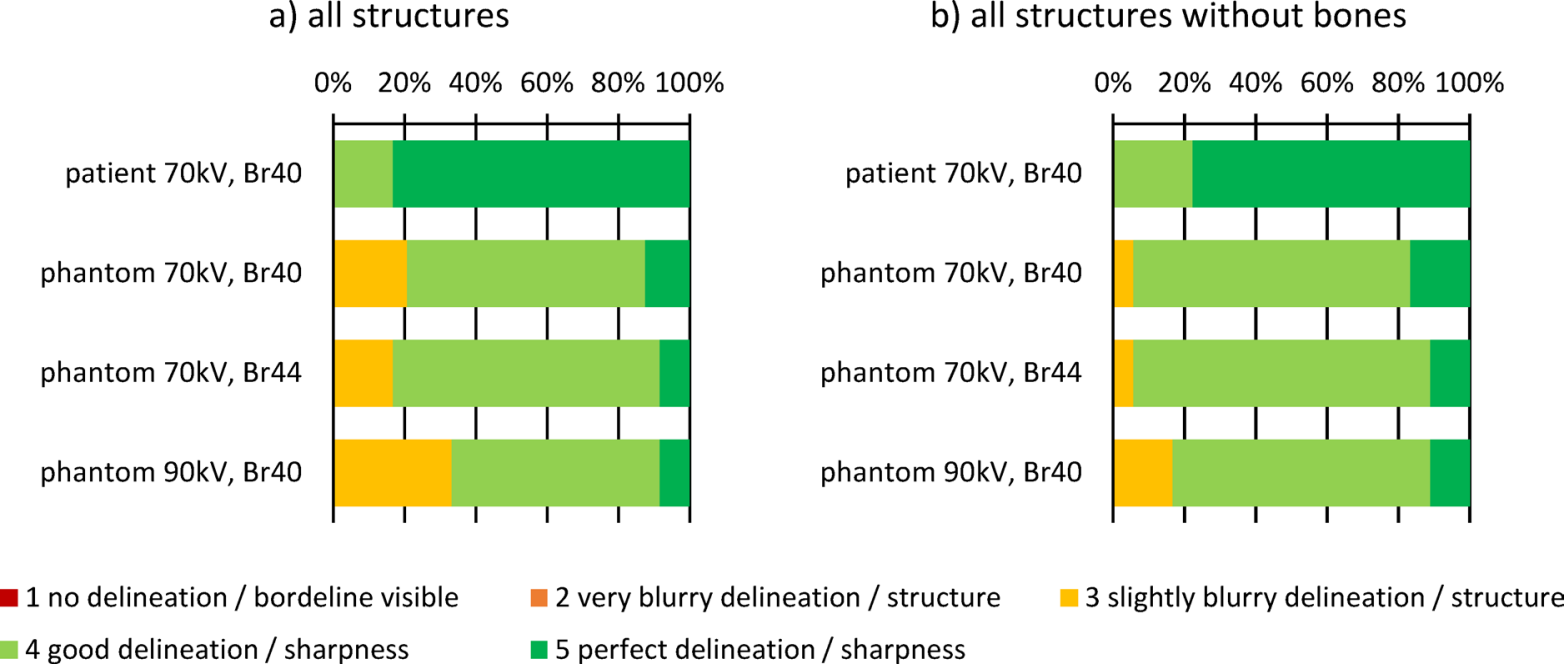
Results of the subjective image quality assessment on a 5-point Likert scale: The stacked bar charts show the subjective ratings in each scan. (**a**) cumulative for the overall image quality of all structures. (**b**) cumulative for all structures, excluding bone structures.

The median, minimum, and maximum ratings on the 5-point Likert scale for each organ are presented in Table 2, and the ratings summarized for (a) all structures and (b) all soft-tissue structures without bones are visualized below (see Fig. 5).

**Table 2:** Evaluation of the subjective image quality rating on a 5-point likert scale: the values median rating [minimum rating; maximum rating] are shown for each scan and reconstruction as well as each organ individually. the patient scan was rated significantly better than the 70 kV Br40 phantom scan (median = 5 vs. 4; *p* < 0.001).

Criteria	Delineation of the					Sharpness of the			
	1) Renal cortex	2) Gallbladder	3) Pancreas	4) Liver hyper-enhancement		5) V. portae branches	6) V. splenica	7) Bone cortex	8) Bone matrix structure
Patient, 70 kV Br40	5 [5;5]	5 [5;5]	5 [4;5]	4 [4;5]		5 [4;5]	5 [5;5]	5 [5;5]	5 [5;5]
Phantom, 70 kV Br40	5 [5;5]	4 [4;4]	4 [4;4]	4 [3;4]		4 [4;4]	4 [4;4]	3 [3;4]	3 [3;4]
Phantom, 70 kV Br44	5 [4;5]	4 [4;4]	4 [4;4]	4 [3;4]		4 [4;4]	4 [4;4]	4 [3;4]	3 [3;4]
Phantom, 90 kV Br40	4 [4;5]	4 [4;5]	4 [3;4]	4 [3;4]		4 [4;4]	4 [3;4]	3 [3;3]	3 [3;4]

**Fig. 6:**
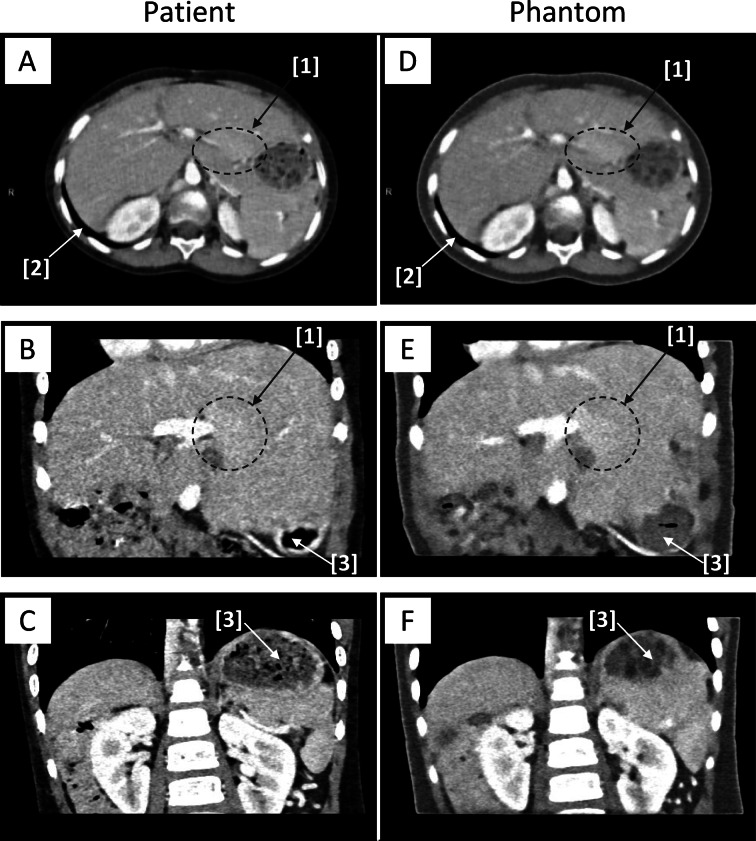
Axial and coronary CT images at W400, C40 of the patient (**A**–**C**; 70 kV, 458 qRef mAs, Br40) and the phantom (**D**–**F**; 70 kV, 461 qRef mAs, Br40) in comparison: An arterial liver hyperenhancement [1] is visible in both the patient and the phantom scan. Air on the phantom’s surface [2] is reproduced accurately, while air inside the torso [3] is mostly printed as solid material. A and D show the delineation of the pancreas and spleen. C and F demonstrate the retroperitoneum and the distinct contrast between the renal cortex and medulla.

The following aspects stand out when comparing the representative image slices of the patient and the phantom scan (70 kV, Br40) shown in Fig. 6. The fat tissue in the phantom is represented at a noticeably lighter gray, i.e., lower CT values. Apart from fat tissue, the CT values appear to be displayed similarly to the patient scan. The phantom exhibits a detailed resemblance to the patient’s anatomy, accurately reproducing small structures, such as peripheral portal venous branches. The air on the surface of the phantom was represented correctly, while the air in the inner phantom (e.g., colon, stomach) was predominantly represented as solid support material. The subjective image quality of the phantom scan appears slightly more blurry; fine structures and organ borders do not appear as sharp as in the patient scan.

## Discussion

In the present study, we demonstrated comparable objective and subjective image quality between the CT scan of a 3D-printed abdominal pediatric phantom and the patient scan when using the same scan parameters.

The phantom demonstrates a high HU-realism in the abdominal structures muscle, liver, pancreas, spleen, portal vein, and aorta (*p* = 0.019). Still, it fails to accurately resemble the relatively high and low radiopacity of fat and bone. The subjective IQ ratings of the parenchymatous abdominal organs, as well as the gray-scale deviations of fat (see Fig. 6), support these findings. The deviations observed on the low-density HU scale (average fat CT values: phantom − 48.3 HU vs. patient − 124.5 HU) are expected due to the limitations of the photopolymer materials. The printer material with the lowest radiopacity (TissueMatrix^®^) has a calibrated CT value of -30 HU, which is too high to accurately reproduce fat in the patient. The observed discrepancy between the mean CT value of fat in the phantom and the theoretical minimal CT value of TissueMatrix^®^ can be attributed to multiple effects. These include potential underestimation of beam hardening and differences in CT spectra due to varying object diameters. The deviations observed at high CT values (average rib bone CT values: phantom 842.5 HU vs. patient 1059.5 HU) are most likely caused by imperfect calibration of the RadioMatrix™ material at 70 kV, due to beam hardening and imperfect spectral calibration. In the future, the utilization of printer materials with a wider range of radiopacities and improved calibration could extend the range of correctly printable CT values.

The phantom scan provides comparable noise levels to those of the patient scan when the same convolution kernels are applied (*p* = 0.027). The overall image impression is slightly blurrier in the phantom scans due to the extensive denoising of the patient scan in preparation for three-dimensional phantom production. In the Br44 reconstruction, which was performed to better compensate for denoising, the image noise increased significantly (*p* < 0.001). However, tissue delineation was only marginally improved.

The phantom exhibited an increased contrast between the portal vein and liver parenchyma, as well as between the aorta and muscle tissue. The squared CNRs were higher than in the patient scan despite a slightly lower CTDI_vol_. This effect may be due to the novel, noise-free reconstruction based on a medium-sharp Qr48 kernel, which avoids systematically printing image noise from the patient scan into the CT Phantom. This denoising process results in a blurring of low-contrast structures and a smoothing effect on the initial images, eliminating all structural information at frequencies above a certain threshold. Consequently, this method causes a higher CNR at a comparable dose. In the reconstruction of the phantom scan, a second smoothing algorithm was applied to the already smooth phantom, resulting in a relative noise level lower than in the patient scan.

The spectral properties of the patient at higher tube voltage (90 kV) are not completely imitated. Instead of showing the expected constant attenuation, the CT values of the parenchymatous organs in the phantom increased with higher tube voltage. This phenomenon, similar to fat, is caused by the photopolymer material Vero^®^ClearB with a calibrated CT value of 100 HU, which is primarily used for parenchymatous organs. This material shows higher CT values at higher tube voltages^36^. The combination of Vero^®^ClearB with a material that exhibits similar calibrated CT values but shows lower CT values at higher tube voltages could potentially solve this issue. In contrast, the attenuation of V. portae, aorta, and ribs, as well as fat, changed as expected.

The phantom displays clinically comparable but significantly lower subjective delineation and sharpness of anatomical structures at comparable qRef mAs (*p* < 0.001). The subjective rating was consistent (ICC = 0.84) and confirms that, while the patient shows a perfect result (median rating 5), the phantom displays a satisfactory delineation and sharpness (median rating 4). The image quality of the phantom is adequate for diagnostic purposes. It performs well in displaying potentially clinically relevant information, e.g., portal venous branches, the contrast between pancreas and spleen, or the delineation of renal cortex and medulla. Remarkable is the comparable conspicuity of the patient’s liver hyper-enhancement in the phantom. This lesion could resemble a hyper-vascular liver pathology in a patient and demonstrates the phantom’s capacity to reproduce low-contrast objects. However, bone structures are not reproduced in the same quality as in the patient scan. The air inside of the patient is not always accurately represented. The support material, which can be dissolved with water after printing, could not be removed in all small cavities, especially in those without connection to the outer surface of the phantom.

The results have demonstrated the phantom’s ability to mimic the subjective image quality, radiodensity, and image noise of the patient scan at comparable parameters, mostly correctly and equivalently. The Br44 reconstruction, performed to compensate for the blurrier image impression because of the denoising, did not result in improved subjective image quality. Future studies should assess the image quality-radiation relationship of the phantom analogous to human tissue with additional scans at varying radiation doses.

Several studies have utilized similar 3D printing technology to create CT phantoms of various structures such as cervical vertebrae^20^, head slices^21,37,38^, breasts^37^, pelvis^39^, lungs^40^, and knees with surrounding soft tissue^20^. However, they evaluated phantoms of other body parts. Most of them were focused on very limited anatomical areas with small ranges of attenuation. No analysis of the image noise as a key parameter of image quality was performed. Previously, CT abdomen phantoms were constructed by gluing together multiple layers of paper printed with iodine-enhanced ink^19,41^. In contrast to these approaches, the 3D printing method proposed herein represents a more practical and automated alternative, allowing for real-size printing. It also leads to less deviation of CT values without the need for gray-scale correction and provides greater mechanical stability. By using an easily removable support material (GelMatrix^®^) in the 3D-printing process, it is, in principle, possible to print internal cavities. This study is the first to utilize photopolymer 3D printing to achieve a realistic representation of abdominal anatomy and image quality for future dose optimizations. Other approaches and studies of constructing abdominal phantoms primarily have a different focus, e.g., dynamic compressibility^42^, a quantitative imaging analysis of selective internal radiation therapy (SIRT) with different lesion inserts^43^, or the simulation of CT-guided procedures^44^. They did not primarily try to achieve an image quality equivalent to that of the patient scan. To our knowledge, this is the first abdomen CT phantom that provides objective and subjective image quality nearly equivalent to that of the patient scan in terms of attenuation and image noise while offering an easy and mechanically stable assembly process.

This study has several limitations, which need mentioning: First, all examinations were performed on only one phantom based on one pediatric patient scanned with a contrast agent and it is unclear if 3D-printing is as exact for bigger patients and native scans. Second, only one PCCT scanner was used, the significance concerning other non-PCCT scanners should be evaluated in the future. Third, the phantom scan was taken at the same IQ level as the patient scan, resulting in similar qRef mAs but an approximately 15% lower CTDI_vol_, possibly caused by positioning the phantom differently from the patient scan^45^. Fourth, further limitations are the number of ROIs in each organ and the number of raters. As the printing technology is relatively new to 3D printing of CT phantoms, its worldwide availability is still limited.

A potential application of the phantom could be to anticipate the image quality-radiation relationship in dose reduction studies, making the examination of real patients obsolete while improving the quality and comparability of those studies. For the medicine-technology industry, anatomically realistic phantoms potentially present an economical and easily reproducible solution that could improve the testing and validation of new CT technology. The phantom could find application in the hands-on training of interventional professionals. It could also help generate analog training data of the same abdomen at different image qualities for AI-based noise reduction in CT.

In conclusion, an abdominal CT phantom was 3D-printed based on the CT anatomy of a pediatric patient, utilizing three photopolymers with varying radiopacities. The results demonstrate comparable objective and subjective image quality to the individual patient at equivalent scan parameters. Future research should prioritize dose reduction analysis to optimize patient safety in computed tomography imaging.

## Data Availability

Datasets generated during this study are available from the corresponding author on reasonable request.
